# A clinical and molecular analysis of *Candidozyma auris* strains from Romania, 2022–2023

**DOI:** 10.1128/spectrum.02809-24

**Published:** 2025-05-19

**Authors:** Marius Surleac, Adriana Mihaela Stanciu, Dragoș Florea, Simona Paraschiv, Daniela Tălăpan, Mirela Flonta, Carmen Cristina Vasile, Gabriel Adrian Popescu, Dan Oțelea

**Affiliations:** 1 Research Institute of the University of Bucharest, University of Bucharest 61783, Bucharest, Romania; 2 “Prof. Dr. Matei Bals” National Institute of Infectious Diseases 277190, Bucharest, Romania; 3 “Carol Davila” University of Medicine and Pharmacy 87267, Bucharest, Romania; 4 Infectious Diseases Clinical Hospital, Cluj Napoca, Romania; University of Natural Resources and Life Sciences Vienna, Vienna, Austria

**Keywords:** *Candida auris*, emergent fungus, healthcare-associated infections

## Abstract

*Candidozyma auris* (formerly *Candida auris*; *C. auris*) raises significant concerns for healthcare facilities due to its ability to cause severe systemic infections, increased resistance to antifungals, and high intra-hospital transmissibility. We conducted a retrospective study on 102 patients diagnosed in 2022 and 2023. Demographic, clinical, and epidemiologic data were collected from the hospital databases. *C. auris* was identified using MALDI-TOF systems, and antifungal susceptibility was determined with Micronaut. The isolates were sequenced using Illumina next-generation sequencing platforms. Molecular epidemiology analysis was performed with multiple bioinformatics approaches (phylogenetic tools, resistance genes-related predictions, and variant analyses). Most patients (80/102) were admitted to or had been recently transferred from intensive care units at the moment of *C. auris* detection. Most cases (57/102) were classified as infections; 29 of them were bloodstream infections. All patients had been treated with broad-spectrum antibiotics before *C. auris* isolation, and 75/102 had received antifungals. All 31 tested isolates showed resistance to fluconazole, 5 were resistant to amphotericin B, and they were all susceptible to echinocandins. Crude mortality for infected patients was 68.18%. The 31 analyzed sequences belonged to clade I and bore multiple resistance markers.

**IMPORTANCE:**

Highly antifungal-resistant *Candidozyma auris* keeps spreading in regions previously free of this pathogen, stressing once again the need for active surveillance, flexible control measures, and antimicrobial stewardship. This study is seminal for our understanding of the *C. auris* outbreak in Romania, providing insights into the evolutionary dynamics and genomic diversity of the pathogen and highlighting clade-specific mutations possibly linked to antifungal resistance. By joining these molecular characteristics with clinical, epidemiological, and microbiology data, such as risk factors for acquiring *C. auris* and phenotypic antifungal susceptibility, the study can be instrumental for surveillance and infection control strategies that are essential due to the pathogen’s high transmissibility and the global health threat that it poses.

## INTRODUCTION

The emerging yeast *Candidozyma auris* is often resistant to antifungal drugs, and it is easily transmissible and able to cause healthcare-associated outbreaks. This fungus can cause severe systemic infections in critically ill patients and is associated with high mortality and increased length of hospital stay. *C. auris* is categorized by the World Health Organization (WHO) as one of the four critical priority fungal pathogens (1). Drug-resistant *C. auris* is classified by the US Centers for Disease Control and Prevention (CDC) as an urgent antimicrobial resistance threat, the first fungus in this highest risk category (2). Furthermore, unlike other *Candida* species, *C. auris* exhibits high thermotolerance and osmotolerance, high resistance to commonly used surface disinfectants, and ability to form biofilms; all these are responsible for prolonged persistence on patients’ skin and mucosa and in the hospital environment, leading to high transmissibility within and between healthcare facilities (3).

*C. auris* has only recently emerged, and its identification and differentiation from closely related species are difficult in settings where biochemical identification methods have not been updated (4).

Since it was first identified in 2009 (5), the simultaneous and apparently independent emergence of several clades has been reported in over 70 countries on 6 continents (6). *C. auris* isolates were initially grouped into four clades by using whole-genome sequencing (WGS) data: clade I (South Asia), clade II (East Asia), clade III (South Africa), and clade IV (South America); a fifth clade and a sixth clade were described in 2018 (Iran) (7) and 2024 (Bangladesh and Singapore) (8).

In Romania, the first *C. auris* cases were reported in the first months of 2022 in a few hospitals in Bucharest that had capabilities for proper identification of this fungus (9). This study aims to describe clinical, epidemiological, and molecular characteristics for some of the first cases and the *C. auris* isolates identified in Romania since 2022.

## RESULTS

During the study period, *C. auris* was detected in 102 patients (demographic data in Table 1). Most patients (58.5%) were admitted to an intensive care unit (ICU) at the time of the *C. auris* detection; the others were hospitalized in a medical or surgical ward (36.6% and 4.9%, respectively). However, half of the non-ICU patients (*n* = 21) had been transferred from an ICU during their respective hospital stay. Their primary diagnosis consisted of severe conditions (Table 1). Most of the patients (89.1%) had underlying comorbidities. The most frequently associated comorbidities were chronic cardiovascular disease (65.6%), type 2 diabetes (34.3%), and neurological disorders (31.3%) (Table 1).

**TABLE 1:** Patient characteristics

	Hospital B	Hospital M	Hospital E	Hospital C	Total
Demographics					
Mean age (years)	64.1	66.7	57	67.5	65.3
Gender (% of men)	73.7%	69%	60%	50%	68.6%
Primary diagnosis					
Infection caused by another pathogen	17	33	6	2	58
Acute neurological conditions	1	18	0	0	19
Acute cardiovascular events	0	5	2	0	7
Malignancy	0	6	0	0	6
Acute gastrointestinal disorders	0	4	0	0	4
Chronic neurological conditions	0	1	1	0	2
Chronic cardiovascular diseases	0	3	0	0	3
Severe traumatic injury	0	1	1	0	2
No data	1	0	0	0	1
Comorbidities					
Chronic cardiovascular disease	12	47	6	0	65
Diabetes mellitus type 2	5	27	2	0	34
Neurological disorders	8	18	5	0	31
Malignancy	7	11	1	0	19
Chronic kidney disease	4	12	2	0	18
Chronic liver disease	3	5	0	0	8
Chronic pulmonary disease	3	5	0	0	8
HIV infection	2	2	0	0	4
No comorbidities	2	6	1	2	11
No data	1	0	0	0	1
Risk factors for *C. auris* presence					
Mean length of stay	47.3	56.8	49.1	N/A^*b*^	54.3
Mean days before first isolation	25.5	35.2	23.7	N/A	32.3
Current or prior ICU admission	16	58	6	N/A	80
Broad-spectrum antibiotics before isolation	18	71	9	N/A	98
Antifungals before isolation	14	53	5	2	74
Concomitant presence of CRE^*a*^	8	5	51	2	66
COVID-19	8	14	1	N/A	23
Total cases	19	71	10	2	102

^*a*^ CRE, carbapenem-resistant *Enterobacterales.*

^*b*^ N/A, no data.

Most of the *C. auris* cases were classified as colonizations (see Table 2). Screening policies were implemented in two of the participating hospitals starting the last trimester of 2022, but the third hospital did not perform screening during the study period. This is likely to induce a bias toward infections and carriages proportion and minimize the number of skin colonizations.

**TABLE 2:** *C. auris* infection and colonization sites^*a*^

	Hospital B	Hospital M	Hospital E	Hospital C	Total
Bloodstream infections	2	14	6	0	22
Colonization	17	45	2	2	66
Urine	4	24	2	1	31
Skin	10	4	0	1	15
Respiratory tract	0	11	0	0	11
Bedsore	2	2	0	0	4
Catheter	1	4	0	0	5
Undetermined	0	12	2	0	14
Total cases	19	71	10	2	102

^*a*^ Twelve of the 66 patients with *C. auris* colonization had multiple colonization sites.

Most patients (16/22) with *C. auris* infection were treated with echinocandins, and only one was treated with amphotericin B. Patients with *C. auris* had prolonged hospitalization, with a mean length of stay of 54.3 days (range 5–202 days, SD = 35.2 days). Positive cultures were obtained after a mean of 32.3 days of hospitalization (range 0–133 days, SD = 20.2 days). All patients for whom data were available (*n* = 98) had been treated with broad-spectrum antibiotics in the previous 90 days before a first positive culture, and 78/98 had received carbapenems. Almost two-thirds of all patients with *C. auris* (65.4%) had concomitant infection or colonization with a carbapenem-resistant *Enterobacterales* species.

A significant proportion of patients (74/102) had also been treated with antifungals in the 90 days before the detection of *C. auris*: 43/102 patients had been previously treated with azoles (41 with fluconazole and 2 with voriconazole), 14/102 patients with echinocandins (usually anidulafungin, two patients received caspofungin), 17/102 patients had received both an azole and an echinocandin, and for 1 patient, the antifungal drug was not mentioned. Another factor associated with *C. auris* presence was corticotherapy: 60/102 patients had been treated with corticosteroids during the 90 days before the first positive culture.

All but four patients were known to have had indwelling medical devices, such as urinary catheters, central and peripheral lines, or nasogastric tubes. Forty-eight patients (47.1%) had a central venous catheter, and 47 of them (46.1%) required endotracheal intubation and mechanical ventilation.

All-cause mortality for patients with a *C. auris* infection was 68.18%, while for patients classified as colonized, it was 60.6%.

None of the patients’ records mentioned any hospital admissions outside Romania.

### Minimum inhibitory concentration values of the *C. auris* isolates

All 31 phenotypically tested isolates were resistant to fluconazole, with minimum inhibitory concentrations (MICs) ranging from 32 to >128 µg/mL, where 5 isolates (16.1%) were also resistant to amphotericin B, with MICs ranging from 2 to 16 µg/mL, but all 31 isolates were susceptible to anidulafungin and micafungin according to the CDC’s tentative MIC breakpoints (10).

### Phylogenetic analysis and clade classification

The 27 available WGS data sets were phylogenetically analyzed with Pathogenwatch; this database contains roughly 2,700 *C*. *auris* genomes. There is a representative reference genome sequence in Pathogenwatch for each of the first five clades. The 27 Romanian *C. auris* genomes were predicted to belong to clade Ib. The clade I set in Pathogenwatch comprises about 1,200 *C*. *auris* genomes; the B13916 genome (GCA_016772235.1) is the reference sequence for clade Ib (11).

**Fig 1:**
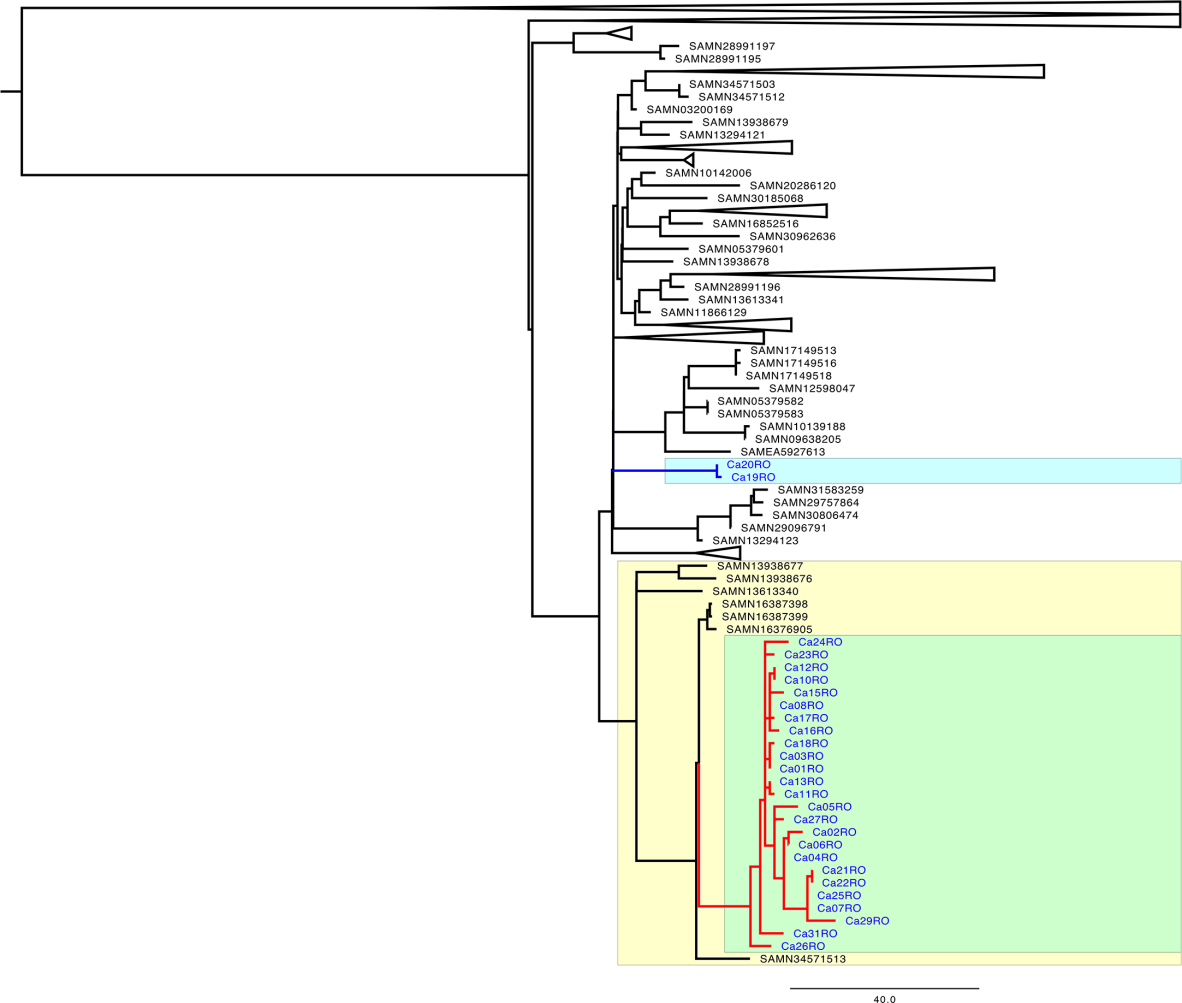
Phylogenetic relationship between Romanian (blue) and other genomes from clade I, according to the Pathogenwatch database.

The 27 Romanian genomes are part of a subcluster consisting of 420 genomes (Fig. 1). One interesting observation is that two Romanian *C. auris* genomes (Ca19 and Ca20) are positioned apart from the rest of Romanian *C. auris* genomes, in a dense group of 354 worldwide genomes (still within the parent 420-genome subcluster). The other 25 Romanian *C. auris* genomes form one well-supported cluster, situated separately on another branch, in the same parent subcluster (Fig. 1). The distinct distribution of Romanian *C. auris* isolates in the phylogenetic tree suggests that there were at least two distinct import events in the country.

The two Romanian isolates (Ca19 and Ca20—highlighted in cyan in Fig. 1), together with genomes clustering separately, were detected in one of the three hospitals from Bucharest and showed phylogenetic relatedness to *C. auris* isolates from several parts of the world (US, Qatar, India, Pakistan, UAE, Germany, Singapore, France, etc.); they are similar to two other close branches made of nine and, respectively, five genomes—from the United States, India, Pakistan, and UAE/The Netherlands/Hong Kong. All the 354 isolates that subcluster together contain the S70R mutation in the *FCY1* gene and the Y132F mutation in the *ERG11* gene, mutations well documented and associated with resistance to nucleoside analogs and azoles, respectively (12, 13).

The remaining 25 Romanian *C. auris* genomes form a well-supported monophyletic cluster (marked in red in Fig. 1), suggesting a distinct introduction of this pathogen in the country, followed by a local spread. Seven other genomes reported between 2015 and 2023 from the United States, India, and France are grouped close to the Romanian cluster. All 32 genomes within this group (highlighted in yellow in Fig. 1) are predicted to be resistant to 5-flucytosine (a drug not in use at the time in Romania) and fluconazole. Another interesting observation is that at the root of the 25-Romanian monophyletic cluster, there is one genome corresponding to one *C. auris* isolate from Cluj Napoca, suggesting that strain might have been among the earliest present in Romania.

All Romanian *C. auris* consensus sequences uploaded to Pathogenwatch are grouped as a set, which includes the phylogenetic analysis in a global context, and it can be accessible on the following link (https://pathogen.watch/collection/guf79k2mfaxw-ca2ndpaper).

### Antifungal resistance-related genes

The sequenced raw reads have been mapped on each antifungal resistance gene listed in the supplemental tables, “Antifungal Resistance*”* sheet. Most of these genes had no synonymous mutations (with few exceptions), while some of them had one or multiple non-synonymous mutations (Table 3). Some of these mutations have been validated in *ERG11* and *FCY1* by comparison with Pathogenwatch. The same non-synonymous mutations have been found in all sequenced Romanian isolates.

**TABLE 3:** Overview of antifungal resistance

Chromosome number	Possible resistance to	Gene	Gene description	Resistance mechanism (if known) based on the literature	References	Non-synonymous substitutions in Romanian isolates
1	Azoles	*MRR1A*	Transcriptional regulator for MDR1	Overexpression of MDR1 due to mutations (clades III and IV)	(14, 15)	*907Y^*a*^
	Echinocandins, azoles	*CIS2*	γ-Glutamyl-transpeptidase, involved in the detoxification of xenobiotics	Mutations	(16)	K74E
2	Azoles	*CDR1*	(*Candida* drug resistance 1) drug exporters of the ABC family	Increased drug efflux due to deletions or overexpressions	(13–16)	E709D
	Polyenes	*ARG1*	Argininosuccinate synthase	Overexpression	(17)	E47K
3	Azoles	*ERG11*	Lanosterol 14 α-demethylase (sterol synthesis pathway)	Reduced affinity to azole due to mutations (e.g., Y132F) or increased gene copy number	(13–16, 18–20)	Y132F
4	Azoles	*MDR1*	(Multidrug resistance 1) drug exporters (efflux pumps) belonging to the major facilitator superfamily	Drug exporters (efflux pump)	(15)	K749R
	Nucleoside analogs (5-flucytosine)	*FCY1*	Cytosine deaminase activity, purine-cytosine permease	Mutations	(14, 19, 20)	S70R
	Polyenes	*ERG2*	Ergosterol synthesis	Overexpression	(12, 14, 16, 19–21)	E39D
5	Azoles	*TAC1b*	Transcriptional regulator for CDR1 (efflux pumps)	Overexpression of CDR1 due to gain-of-function mutations (e.g., A657F) or deletions; independent mechanism?	(14, 16, 19)	K691E
		*HSP90* *-STI1*	Heat shock protein (mediator of tolerance)		(15, 20)	A35V, K66E, L260S, H349Y
6		*CDR2*	Drug exporters of ABC family	Overexpression	(13, 16, 20)	M67T, C76S, P129L
7	Polyenes	*FAS2*	Fatty acid biosynthesis		(17)	E1211D
		*STE6*	ABC family transporter		(12)	G240S, D421--, K1218N

^*a*^ *, stop codon.

### Sequence variant analysis and annotations

The sequence variant analysis performed on all Romanian *C. auris* genomic sequences from this study shows a homogeneous chromosomal distribution of variants among the isolates, suggesting close relatedness among the sequences of the 25-Romanian monophyletic cluster. One of the Romanian genomes, namely Ca26, which was isolated in a hospital from Cluj Napoca (a city in north-western Romania), has the lowest number of variants throughout the genome (see the supplemental tables, “Variants_Annot_by_Chr*”* sheet). Similar to the Ca26 isolate, there is another isolate from Bucharest, namely Ca31, with a low number of sequence variants on each chromosome. These two isolates are placed at the root of the monophyletic Romanian cluster and may have contributed to the initial spreading of this pathogen within the country.

The highest number of sequence variants (*n* = 174 in average) was found on chromosome 1, and the average numbers decrease with increasing numbering of the chromosomes: 113 variants on chromosome 2, 86 variants on chromosome 3, 71 variants on chromosome 4, 55 variants on chromosome 5, 49 variants on chromosome 6, and 31 variants on chromosome 7. Most sequence variants are represented by insertions (between 62% and 75% on average throughout the chromosomes) and by single-nucleotide polymorphisms (SNPs; between 17% and 29% on average throughout the chromosomes), followed by deletions and complex variants.

Based on the annotations, we noticed that some CDS genes appear multiple times (up to 10 times) throughout the genome. Multiple copies were usually on the same chromosome but on different chromosomes as well (see the supplemental tables, “CNV_by_Annot*”* sheet). Most of these genes are found on chromosome 1, while the lowest number is found on chromosome 5.

## DISCUSSION

*C. auris* is an emergent healthcare problem in several countries, Romania among them. The European Centre for Disease Prevention and Control (ECDC) has issued the first rapid risk assessment report in 2016 aiming to raise awareness of the risk of *C. auris* spread in hospitals from the European Union and the European Economic Area countries; two updates in 2018 and 2022 followed (22).

Ten European countries have detected *C. auris* cases according to the 2022 ECDC report (22). Romania was not included in this report because the first cases were only identified at the beginning of 2022 (9).

Here, we present an analysis of all the *C. auris* cases in three hospitals in Bucharest and one in Cluj, describing clinical and epidemiological features as well as the genomic and phenotypic antifungal susceptibility data available.

The risk factors identified in the *C. auris* patients were similar to those associated with other *Candida* species (and opportunistic pathogens in general): concomitant severe conditions, immunosuppression, indwelling medical devices, prolonged hospitalization and ICU stay, and exposure to broad-spectrum antibiotics and antifungals. These are consistent findings reported by other groups (23). Notably, a high percentage of the patients from this study had concomitant carbapenem-resistant *Enterobacterales* species colonization. This observation emphasizes the importance of antimicrobial stewardship in reducing not only the risk of acquiring multidrug-resistant bacteria but also that of invasive fungal infections.

Invasive infections with *Candida* species have historically been associated with hospitalization in the ICU (24), and *C. auris* is no exception. Moreover, due to the distinct ability to persist on surfaces and intra-hospital transmission, the correlation between *C. auris* carriage or infection and hospitalization both in the ICU and in non-ICU wards was found to be very strong by the current study and by other reports as well (23, 25).

The proportion of symptomatic infections in our study was significantly higher than those identified by other studies (26). This difference can be explained by the fact that *C. auris* colonization systematic screening had been implemented in two of the three participating hospitals in 2022, and it started in October 2022. Given the novelty of the pathogen, a more aggressive approach was adopted by the physicians when facing a positive *C. auris* specimen, and, therefore, an overestimation of the infection count in this study.

*C. auris* infections are known to be life-threatening, particularly the invasive ones that tend to occur in immunodeficient patients. Crude mortality rates vary greatly in literature, between 27% and 70% (22, 26), but attributable mortality has yet to be accurately determined. The high mortality identified in colonized patients in our study, as well as in other studies (27), is probably due to the underlying diseases.

*C. auris* is a high matter of concern due to its resistance to one or more antifungals (26). Antifungal susceptibility testing for *C. auris* is difficult since neither the European Committee for Antimicrobial Susceptibility Testing (EUCAST) nor the Clinical and Laboratory Standards Institute has established MIC breakpoints, but, in the interim, the CDC has established tentative resistance breakpoints based on those existing for other *Candida* species (10). Most *C. auris* strains are resistant to fluconazole, while resistance to amphotericin B is below 50%, and echinocandins generally remain active but with higher MICs than those for *Candida albicans* (26, 28). One study described that *C. auris* resistance to antifungal drugs is variable not only by drug but also by clade. The multidrug resistance (MDR), defined as resistance to two major antifungal classes, is described to be the highest in clade I isolates (18). This pattern was also noticed in the current study: all phenotypically tested isolates were resistant to fluconazole, 16% were MDR (resistant to fluconazole and amphotericin B), and none was resistant to anidulafungin and micafungin.

Resistance is probably multifactorial, and multiple mechanisms have been described: drug target alterations (i.e., mutations in the *ERG11* gene encoding the main enzyme targeted by azoles), overexpression of *ERG11,* and overexpression of efflux pumps. A major mechanism for azole resistance in *Candida* species, including *C. auris*, is based on substitutions in the *ERG11* gene. The most commonly described substitution in clade I is Y132F, also identified in all fluconazole-resistant sequenced isolates from the current study.

Overexpression of efflux pumps is another mechanism contributing to azole resistance in several *Candida* species, including *C. auris*. The most described efflux-pump-encoding genes are *CDR1* (*Candida* drug resistance 1), *CDR2,* and *MDR1* (multidrug resistance 1). Overexpression is predominantly due to mutations in *TAC1B* and *MRR1A*, the transcriptional factors for *CDR1/CDR2* and *MDR1,* respectively (29). Some of the most frequently described mutations associated with fluconazole resistance in clade I isolates are A640V, A657V, F214S, or R495G in *TAC1B* and N647T in *MRR1A* (30). All sequenced isolates from the current study had substitution E709D in the *CDR1* gene, a previously described variant possibly associated with azole resistance (12), and three novel variants, K749R in *MDR1*, K691E in *TAC1B,* and *907Y in *MRR1A* (Table 3). The significance of these variants remains to be determined in further *in silico* and experimental studies, especially since the role of *MDR1*, belonging to the major facilitator superfamily, and its transcriptional regulator on fluconazole resistance was demonstrated in clade III isolates only (30).

Resistance to amphotericin B is less understood: The most documented mechanisms are overexpression of genes involved in the ergosterol biosynthesis (*ERG1*, *ERG2*, *ERG3*, *ERG5*, *ERG6*, and *ERG13*) or drug target alterations due to mutations in *ERG2*, *ERG6,* or the transcriptional factor *FLO8* (16, 19, 30, 31). Mutation E39D in the *ERG2* gene was found in all isolates from the current study; although described in a few resistant clade I isolates (12, 21, 32), its significance to polyene resistance seems questionable. Existing data about mutations in the *CIS2* gene are conflicting: one study (12) did not find a correlation between this gene and antifungal resistance, while another recent one (13) has found an association between *CIS2* and echinocandin resistance. The other non-synonymous mutations from the other genes that are listed in Table 3 have not been mentioned in the literature. Therefore, these could represent novel mutations associated with the Romanian *C. auris* isolates; their impact, if any, on resistance is yet to be determined.

Phylogenetic analysis suggested two possible imported events in this country. The larger transmission cluster consists of 25 Romanian genomes. The variant analysis shows an increased homogeneity among these genomes. This observation combined with phylogenetic clustering is an indication of local dispersion after the first introduction of *C. auris* in Romania. Sequence Ca26 from Cluj-Napoca is present at the root of this cluster, suggesting it being among the first present in this country. In addition to this hypothesis, SNP data show that the Ca26 genome has the lowest number of variants compared to the reference sequence (see the supplemental tables, “Variants_Annot_by_Chr*”* sheet). The closest foreign genome to the Romanian large cluster was described in the United States in 2023; two more distant ones were also described in the United States in 2020.

## MATERIALS AND METHODS

### Patient population

We conducted a retrospective observational study spreading over a 24-month period, from January 2022 to December 2023. All patients from the participating hospitals who had *C. auris* infection or colonization were included in the analysis. The majority of the isolates (98/102) were identified in the Bucharest-Ilfov area from the only three hospitals that had adequate diagnostic tools to identify *C. auris* and communicated the cases. Later, in the study period, four more isolates from outside the Bucharest-Ilfov area became available for phenotypic and molecular testing and were included in the study in order to expand the geographical coverage. The data collected included demographics, primary diagnosis, comorbidities, risk factors for *C. auris* infection or colonization, whether the *C. auris* identification was considered an infection or colonization, and the antifungal treatment administered in the case of infection.

The *C. auris* presence was considered an infection if the specimen was collected from a normal sterile site or if the attending physician considered it to be an infection based on symptoms and laboratory findings and if antifungal treatment was administered. However, urine and respiratory samples were classified as colonizations, considering the fact that yeast infections in these sites are very rare and require extensive diagnostic tests for confirmation, which were not available in this retrospective study (22, 26). Bloodstream infection diagnosis was established based on positive peripheral blood samples and not catheter blood. Positive catheter samples in the absence of positive peripheral blood samples were interpreted as catheter colonization or undetermined.

When analyzing the risk factors for acquiring a *C. auris* infection or colonization, exposure to broad-spectrum antibiotics was defined as treatment with an antibiotic from the Watch or the Reserve group of the WHO’s AWaRe classification for antibiotics in the previous 90 days before the first positive sample (33).

### Fungal isolates

The *C. auris* isolates from all 102 patients were identified using a MALDI-TOF Biotyper (software version 3.1, Bruker Daltonics GmbH & KG, Bremen, Germany), in accordance with the Centers for Disease Control recommendations (34). Sample preparation was carried out following the manufacturer’s instructions: a thin smear of yeast biomass was placed onto a target plate, then treated with 1 µL of 70% formic acid, then it was overlaid with 1 µL of alpha-cyano-4-hydroxycinnamic acid matrix solution (Bruker Daltonics, Germany), and prepared according to the manufacturer’s protocol. The cut-off value for species identification (log) score was 1.7, as this threshold is considered to be optimal (35).

Antifungal susceptibility was determined using broth microdilution micronaut-AM plates (Bruker Daltonics GmbH & KG, Bremen, Germany) for the available 31 isolates. The antifungal susceptibility results were interpreted using the CDC tentative minimum inhibitory concentration breakpoints (10). In accordance with the EUCAST recommendations, isolates that are susceptible to anidulafungin and micafungin were also considered susceptible to caspofungin (36).

### Whole-genome sequencing

*C. auris* isolates cultured overnight on brain-heart infusion broth were pelleted and processed for nucleic acid extraction using reagents included in the Ultra Clean Microbial Kit (Qiagen). We have used the Illumina DNA Prep Kit and Integrated DNA Technologies (IDT) for Illumina DNA/RNA unique dual (UD) indexes for library preparation. Full-genome sequences were successfully generated for 27 of the 31 strains that had been tested previously for phenotypic resistance.

### Bioinformatic analysis

The sequenced raw reads have been initially polished, trimmed, and *de novo* assembled using the *shovill* pipeline with SPADES 3.12.067. The resulting contigs were further used for BLAST reference sequences search. The *C. auris* sequences underwent reference mapping with Geneious Prime 2023.0.1. The resulting consensus sequences for each of the sequenced strains were further subjected to clade classification by mapping them onto clade-specific sequences, which were downloaded from the NCBI database as previously described (9). The raw reads have been mapped against 42 antifungal resistance genes (see the supplemental tables, “Antifungal Resistance*”* sheet) from NCBI and Candida Genome Database (37).

The whole-genome consensus sequences obtained with Geneious Prime were further compared with the Pathogenwatch database (38) to double-check clade classification and antifungal resistance genes. The raw reads have been QC filtered again using the Trimmomatic (39) and HTStream (40) tools, and then the resulting output has been subject to reference mapping and variant analysis with Snippy (9). The reference used for Snippy was the clade I reference that resulted from the Pathogenwatch analysis, namely B13916, which corresponds to clade Ib (11). Phylogenetic analysis was carried out using Pathogenwatch on the whole genomes of *C. auris* isolates. Because the B13916 reference is not annotated in NCBI, its annotation has been assigned in Geneious Prime by analogy with the B11205 reference sequence—it belongs to clade Ic (11), and it is the closest to B13916. Therefore, variant analysis also included the annotations on each chromosome. A copy number has also been assigned to the reference sequence and to the isolates in this study based on the existing annotations.

### Study limitations

Due to the retrospective data collection, some clinical and epidemiological patient information could not be accessed, and not all *C. auris* isolates were available for antifungal susceptibility testing. Another limitation is the difference in the screening policies implemented in the participating hospitals during the studied period, with probable impact on the proportions of *C. auris* carriages. All this considered, the study is not representative, and it was not intended to be; it rather gathered all information (inevitably incomplete) and strains at the early stages of *C. auris* detection in Romania; this is why, for instance, only a limited number of the identified strains were also available for sequencing. Further studies are definitely needed to detail and follow the evolution of the epidemic. There are also some bioinformatic limitations to this study. One of them is given by the limited precision of the analysis due to insufficient functional annotation of the genomes in the major databases.

### Conclusions

Strains of *C. auris* are highly resistant to treatment, some with distinct molecular characteristics, keep spreading in regions previously free of this pathogen.

## Data Availability

The data that support the findings in this study are available from the corresponding author upon reasonable request. The WGS raw data generated in this study were submitted to the NCBI database and have the following accession numbers: SRX20872307-SRX20872322 (PRJNA991119), SRX22339644-SRX22339650, SRX25274814-SRX25274817 (PRJNA1035021). All Romanian *C. auris* consensus sequences have been uploaded to Pathogenwatch and are accessible at https://pathogen.watch/collection/guf79k2mfaxw-ca2ndpaper.

## SUPPLEMENTAL MATERIAL

Supplemental material spectrum.02809-24-s0001.xlsx Tables S1 to S4.

ASM does not own the copyrights to Supplemental Material that may be linked to, or accessed through, an article. The authors have granted ASM a non-exclusive, world-wide license to publish the Supplemental Material files. Please contact the corresponding author directly for reuse.
